# Optimization and validation of cryostat temperature conditions for trans-reflectance mode FTIR microspectroscopic imaging of biological tissues

**DOI:** 10.1016/j.mex.2017.01.006

**Published:** 2017-02-02

**Authors:** Sumedha Liyanage, Rohan S. Dassanayake, Amal Bouyanfif, Erandathi Rajakaruna, Latha Ramalingam, Naima Moustaid-Moussa, Noureddine Abidi

**Affiliations:** a Fiber and Biopolymer Research Institute, Department of Plant and Soil Science, Texas Tech University, Lubbock, TX 79403, USA; b Department of Nutritional Sciences, Texas Tech University, Lubbock, TX 79409, USA; c Obesity Research Cluster, Texas Tech University, Lubbock, TX 79409, USA

**Keywords:** Cryo-sectioning and FTIR microspectroscopic imaging of biological tissues, Cryostat, FTIR, Microspectroscopic imaging, Tissue sectioning

## Abstract

In Fourier transform infrared (FTIR) microspectrocopy, the tissue preparation method is crucial, especially how the tissue is cryo-sectioned prior to the imaging requires special consideration. Having a temperature difference between the cutting blade and the specimen holder of the cryostat greatly affects the quality of the sections. Therefore, we have developed an optimal protocol for cryo-sectioning of biological tissues by varying the temperature of both the cutting blade and the specimen holder. Using this protocol, we successfully cryo-sectioned four different difficult-to-section tissues including white adipose tissue (WAT), brown adipose tissue (BAT), lung, and liver. The optimal temperatures that required to be maintained at the cutting blade and the specimen holder for the cryo-sectioning of WAT, BAT, lung, and liver are (−25, −20 °C), (−25, −20 °C), (−17, −13 °C) and (−15, −5 °C), respectively. The optimized protocol developed in this study produced high quality cryo-sections with sample thickness of 8–10 μm, as well as high quality trans-reflectance mode FTIR microspectroscopic images for the tissue sections.

• Use of cryostat technique to make thin sections of biological samples for FTIR microspectroscopy imaging.
• Optimized cryostat temperature conditions by varying the temperatures at the cutting blade and specimen holder to obtain high quality sections of difficult-to-handle tissues.
• FTIR imaging is used to obtain chemical information from cryo-sectioned samples with no interference of the conventional paraffin-embedding agent and chemicals.

## Graphical abstract

**Figure:**
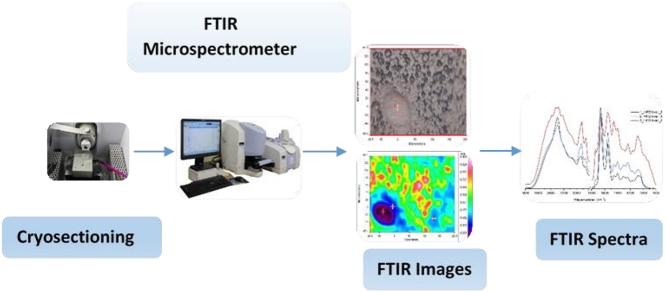

## Method details

### Protocol background

The purpose of this study is to recognize artifacts on the sections as they are being sectioned,optimize the cryostat temperature conditions for different biological tissues (which are robust to section), and validate the protocol with trans-reflectance mode FTIR microspectroscopic imaging. Tissues with high fat content (adipocytes), hard tissues (collagenous tissues such as cervix, scalp), cavernous tissues (such as heart and liver) and lung tissues are extremely difficult to section with desirable thicknesses due to curling and shattering [1]. One of the most important variables to obtain quality frozen sections is maintaining the correct temperature of the sample block that needs to be sectioned. When the temperature of the sample block is very warm, sectioning of the tissue is at a risk of being chunked out completely from the sample holder as the sample block and embedding medium has not hardened enough. Very warm tissue can also crumple completely as the tissue is not firm enough to sustain a flat paper-like shape. As the temperature cools closer to the correct cutting temperature, sections harden and flow over the cutting blade in complete sections. However, sections can still slightly crumple at this stage. As the temperature continues to drop and reaches the ideal temperature, the tissue becomes hard enough to maintain its shape and it is shaved from the sample block with a flat-paper like shape. Therefore, choosing the ideal cutting temperature condition in the cryostat is crucial in order to obtain complete tissue sections with minimal curling and shattering.

### Materials and methods

Previously frozen tissue samples of lung, liver, WAT and BAT from mice fed high-fat diets were used. Tissues were collected under a protocol approved by the Animal Care and Use Committee of Texas Tech University [2]. Tissues were snap frozen for further analyses. Harvested tissues were stored in a Locator 6 Rack and Box System filled with liquid nitrogen below −180 °C prior to sectioning. Tissue samples were cut into ∼0.5 cm in any one dimension and placed in a cryomold (Tissue-Tek Cryomold, Sakura Finetek, USA Inc) half-filled with Optimal Cutting Temperature (OCT) compound (Tissue-Tek OCT, Ted Pella Inc, CA, USA). Following which, the tissue was completely embedded in OCT. Cryomold containing the tissue sample in OCT was kept in the cryostat (RMC, CRT 900, Boeckeler Instruments Inc, AZ, USA) at −20 °C and left for 180 s until the OCT compound was completely frozen. Then, the frozen tissue block was removed from the cryomold and mounted onto the specimen holder. In order to obtain better sections, the temperature at both the blade stage and the specimen holder were optimized in the cryostat chamber. Table 1 summarizes the optimal temperature conditions maintained at the cutting blade and specimen holder for different tissues. The tissue blocks were allowed to equilibrate in the cryostat chamber for 30 min as shown in Table 1. The sections were cut with the cryostat until the region of interest was reached and then series of sections of the tissue sample were taken at desired thickness. The tissues were sectioned with 8–10 μm thicknesses to obtain better trans-reflection mode FTIR spectra of the tissue samples. To ensure a high degree of homogeneity, sections were immediately transferred to a room temperature low-e microscopic slide (MirrIR, Kelvey Technologies, OH, USA) by touching the slide to the tissue. This protocol was followed three times for all tissues. Importantly, high-quality sections were obtained at all attempts. Finally, the sections were kept in a desiccator with a vacuum pump in a cold room overnight. Before imaging, each slide was brought to room temperature in a dark slide box and the sample exposure to light was kept minimal before data collection.

**Table 1:** The optimal temperature conditions maintained at the cutting blade and the specimen holder for different biological tissues.

Tissue Sample	Thickness (μm)	Temperature at the cutting blade (°C)	Temperature at the specimen holder (°C)
WAT	8–10	−25	−20
BAT	8–10	−25	−20
Lung	8–10	−17	−13
Liver	8–10	−15	−5

**Fig. 1:**
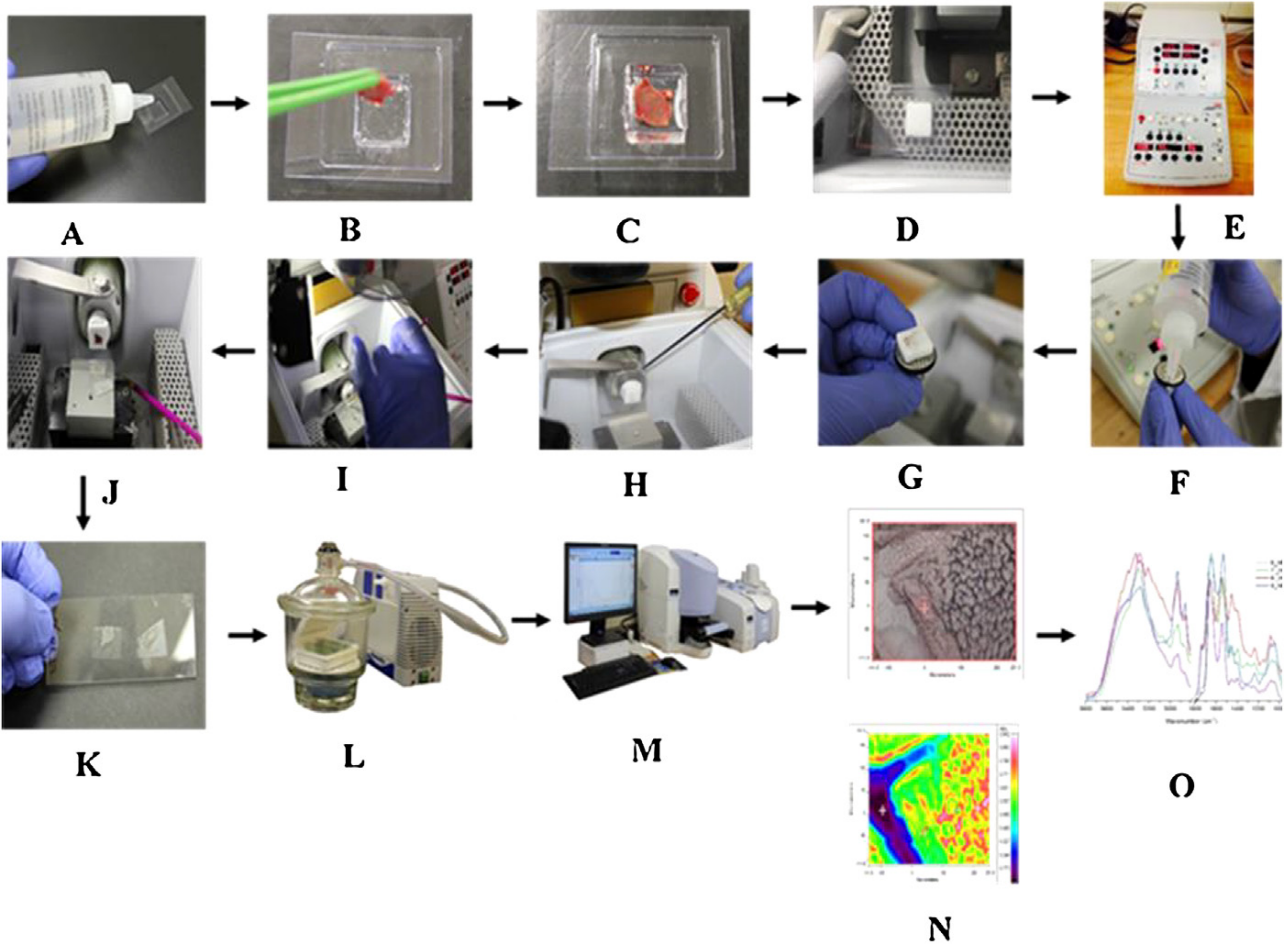
Schematic representation of the overall experimental workflow for the tissue imaging. For each tissue sample (A) cryomold was half-filled with OCT, (B,C) tissue sample was placed in the cryomold and covered with OCT, (D) cryomold was placed inside the cryostat for 3 min until completely frozen, (E) temperature at the cutting blade and specimen holders was adjusted to optimal values (F,G,H) specimen block was removed from the cryomold and placed on the specialized metal grid that fit onto the specimen holder, (I,J) sections were cut in the cryostat, (K) sections were transferred to a low-e microscopic slide, (L) before imaging, sections were kept in a desiccator and dried under vacuum pump, (M,N) IR spectra were recorded on the tissue sections and (O) respective spectral data were obtained.

Controlled experiments were also employed to obtain sections from all tissues by maintaining a constant cryostat temperature of −23 °C for liver sections and −25 °C for WAT, BAT and lung sections as reported in the literature [3], [4], [5], [6]. However, these attempts were unsuccessful and high quality sections were not obtained with high repeatability. An illustration of the overall experimental workflow for the cryo-sectioning is depicted in Fig. 1.

### FTIR microspectroscopy of biological tissues

FTIR images were obtained using a PerkinElmer spectrum Spotlight 400 imaging system, equipped with an IR microscope. The Spotlight ATR imaging accessory employs a Germanium crystal with a high refractive index (∼4.0). The Spotlight 400 is equipped with Liquid N_2_ cooled detector Array Mercury-Cadmium-Telluride MCT, movable and software controlled x, y stage. It can operate in reflection or transmission modes (4000–720 cm^−1^). Images were collected in trans-reflectance mode at a spatial resolution of 16 cm^−1^ in the wavenumber region between 4000 and 750 cm^−1^ with 6.25 × 6.25 μm IR detector pixel size and 128 scan numbers per pixel. The background spectra was collected from an area free of tissue of the low-e glass slide and subtracted automatically from tissue spectra by the use of Spotlight Autoimage software (Perking Elmer Instruments, Boston, MA, USA).

## Method validation

### FTIR microspectroscopic imaging of biological tissues

**Fig. 2:**
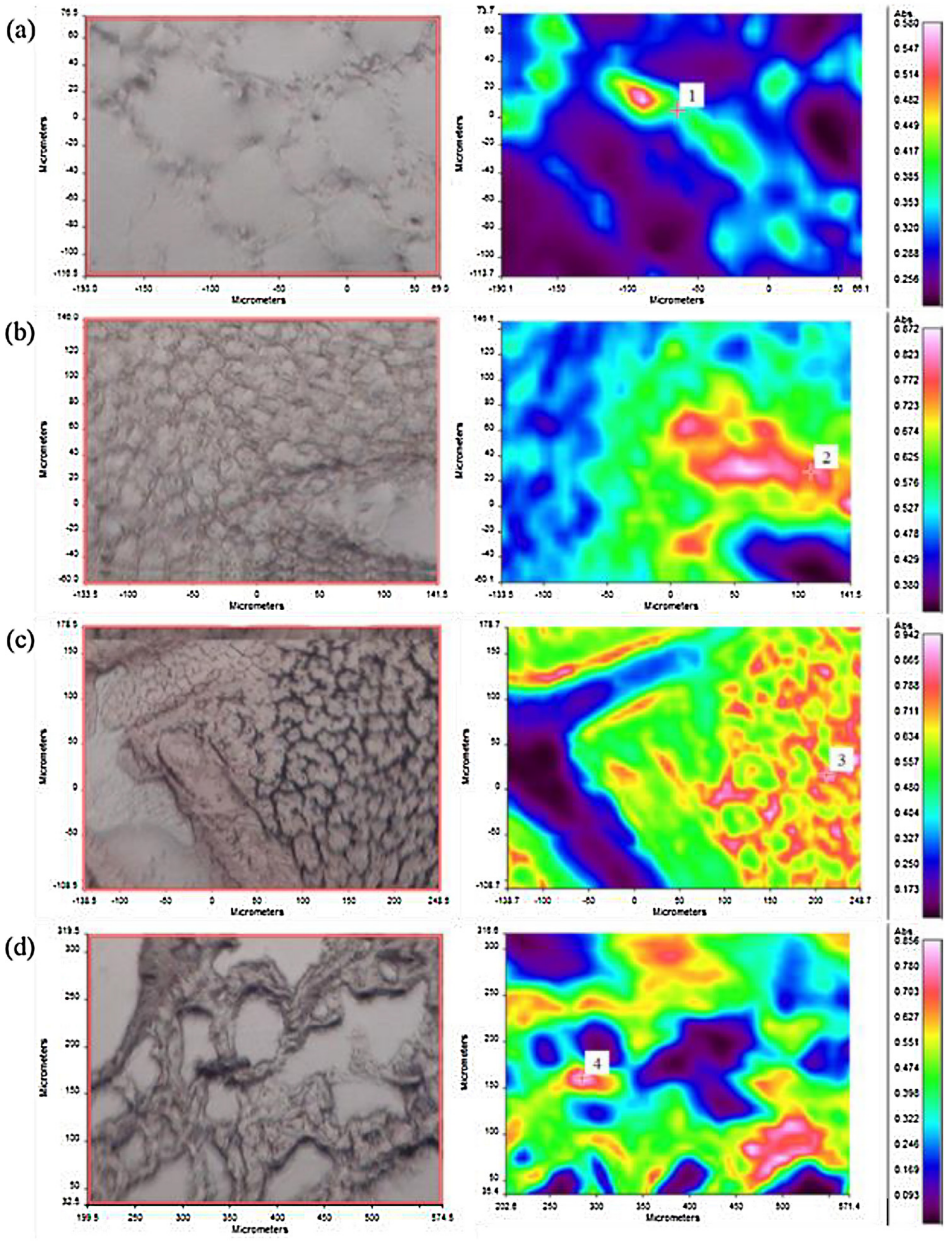
FTIR visual images and the corrosponding average absorbance images of (a) WAT, (b) BAT, (c) Liver and (d) Lung, recpectively. The red color represents areas of the highest absorbance while the blue color represents areas with the lowest absorbance.

In order to validate this method, we imaged all the tissue sections using the trans-reflectance mode FTIR microspectroscopic imaging. Biological tissue samples contain different biochemical components including proteins, lipids, carbohydrates and nucleic acids. All of these biomolecules have their specific vibrational fingerprints which enable FTIR microspectroscopic imaging to obtain visual images of the individual tissue where each pixel is composed of a spectrum originating from vibrational fingerprints [4], [7], [8]. Fig. 2 shows representative FTIR visual images and the corresponding false color average absorbance images of the tissues sections of (a) WAT, (b) BAT, (c) liver and (d) lung in the 4000–750 cm^−1^ region. False color scale runs from high absorbance (red) to low or absent (blue).

### Data analysis

**Fig. 3:**
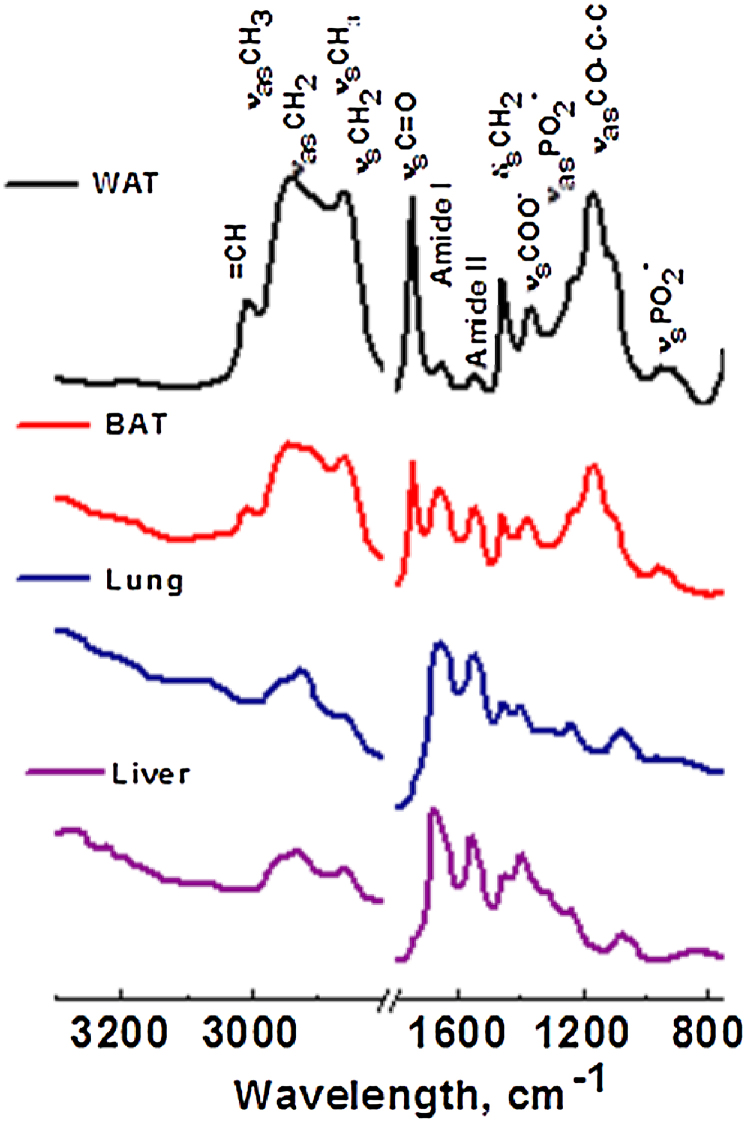
Average trans-reflectance FTIR spectra of WAT (1), BAT (2), Lung (3) and Liver (4) tissue sections (10 μm thickness) in the 3300–2800 and 1800–750 cm^−1^ regions revealing the distinct spectral regions for lipids, proteins, nucleic acids, and carbohydrates. ν = stretching vibrations, δ = bending vibrations, s = symmetric vibrations and as = asymmetric vibrations.

Fig. 3 represents the average absorbance spectra for the tissue sections with 10 μm thickness from WAT, BAT, lung, and liver, respectively in the 3300-2800 and 1800–750 cm^−1^ regions. This comparison shows a general spectral profile of the major peaks originating from biocomponents in each of the tissue section. Fig. 3 shows major absorption features for lipids, proteins, nucleic acids and carbohydrates.

**Fig. 4:**
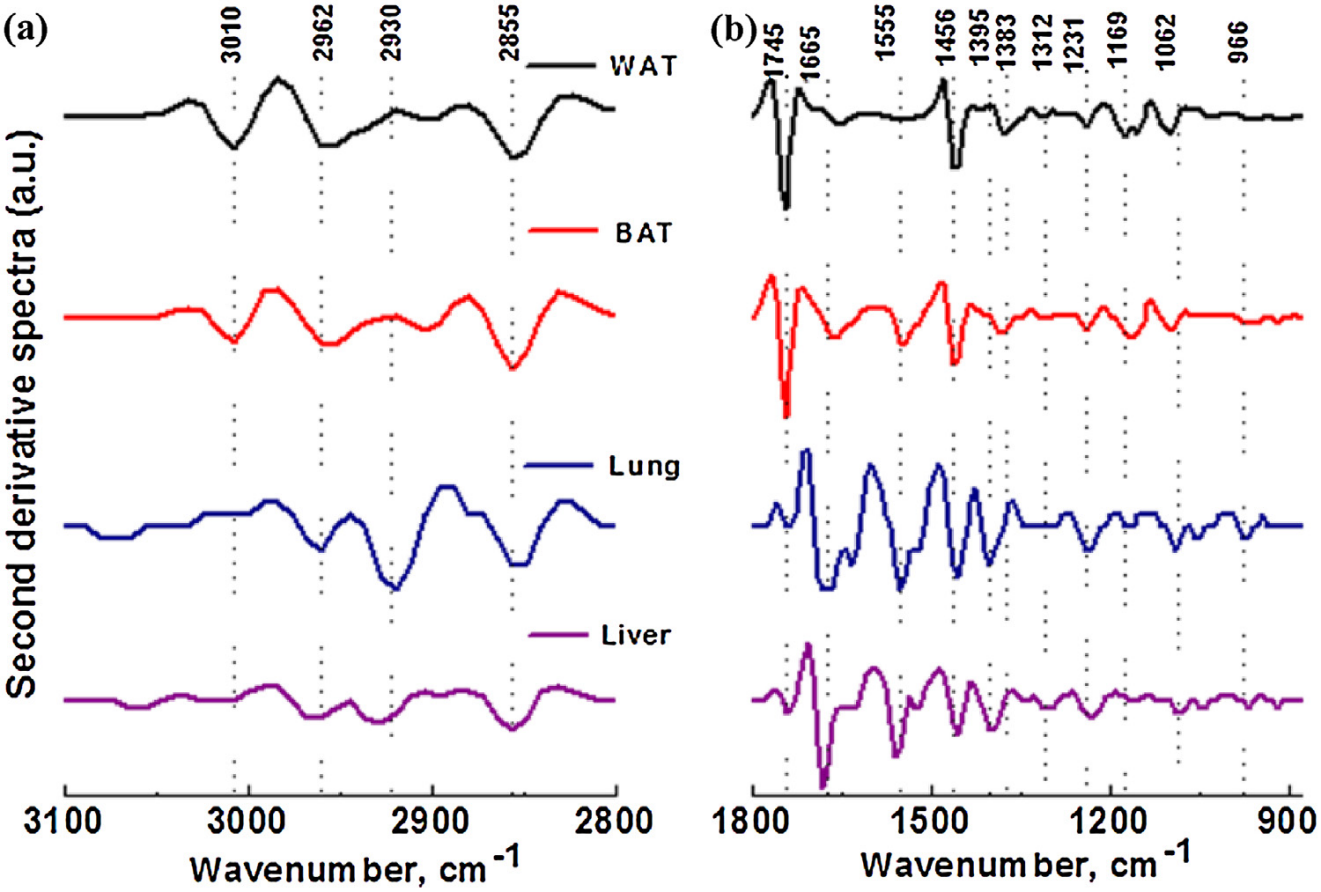
Second derivative FTIR spectra generated from Fig. 2 of liver, lung, BAT and WAT tissue sections (10 μm thickness) in the regions of (a) 310–2800 cm^−1^ and (b) 1800–875 cm^−1^.

Fig. 4 shows the second derivative spectra for tissue sections with 10 μm thickness from liver, lung, BAT and WAT in the regions of 3100–2800 cm^−1^ and 1800–875 cm^−1^. The second derivative spectra provide small differences in spectral band shapes and show major absorption features for lipids, proteins, nucleic acids, and carbohydrates. This is widely used to remove the baseline contribution and to obtain resolving bands, which are often broad and overlapped. Table 2 summarizes the peak wavenumber values and their assignments observed in the FTIR spectra of tissues investigated.

**Table 2:** General assignment of frequency to chemical functions observed in FTIR spectra of the tissues studied [7], [8], [9], [10], [11], [12].

Wavenumber (cm^−1^)	Chemical function		Assignment
∼3290	N—H stretch (amide A)		Proteins
3060	N—H stretch (amide B)	Proteins	
3004–3010	=C—H olefinic stretch	Unsaturated lipids, triglycerides, fatty acids	
2956–2962	CH_3_ asymmetric stretch	proteins, lipids, triglycerides	
2910–2930	CH_2_ asymmetric stretch	Mainly saturated lipids, proteins, triglycerides	
2870	CH_3_ symmetric stretch	Mainly proteins, lipids, triglycerides	
2849–2855	CH_2_ symmetric stretch	Mainly lipids, proteins, triglycerides, fatty acids	
1742–1746	Carbonyl C=O stretch	Triglycerides, phospholipids, cholesterol esters	
1735–1736	Carbonyl C=O stretch	Triglycerides, phospholipids, cholesterol esters	
1680–1710	Carbonyl C=O stretch	Free fatty acids, nucleic acids (DNA)	
1670–1695	Carbonyl C=O stretch + N—H bend (amide I)	Proteins (anti-parallel β-pleated sheet and β-turns structures)	
1648–1670	Amide I	Proteins (α-helix structure)	
1623–1637	Amide I	Proteins (β-pleated sheet structures)	
1550–1555	N—H bend + C—N stretch (amide II)	Proteins	
1539–1546	Amide II	Proteins	
1515–1520	Ring C—C stretch	Tyrosine proteins	
1456–1463	CH_2_ bending mode	Mainly lipids, proteins and cholesterol esters	
1449–1455	CH_2_, CH_3_ deformation modes	Mainly Proteins, lipids	
1383–1395	COO^−^ symmetric stretch	Fatty acids, amino acids	
1373–1379	CH_3_ deformation of aliphatic of amino acid residues	Proteins	
1343	CH_2_ wagging	Phospholipids, triglycerides, fatty acids, amino acid side chains	
1340–1312	Amide III	Proteins	
1231–1234	PO_2_^−^ symmetric stretch	Nucleic acids (DNA), phospholipids, amide III, phosphorylated proteins	
1158–1173	CO—O—C asymmetric stretch	Cholesteryl esters, phospholipids	
1150	C—O stretch	Glycogen, mucin	
1124–1127	C—O stretch	Polysaccharide, lactate	
1120	C—O stretch of ribose ring	Nucleic acids (RNA)	
1086–1097	PO_2_^−^ asymmetric stretch	Nucleic acids (DNA, RNA), phospholipids, glycolipids	
1080	C—C stretch	Glycogen	
1063	−CO—O—C stretches	Phospholipids, cholesterol esters	
1050–1056	COH deformation	Mucin, carbohydrates	
1030	COH deformation	Nucleic acids	
1022–1028	COH deformation	Glycogen, carbohydrates	
970–976	Dianionic phosphate monoester	Phosphorylated proteins, phospholipids	
965	C—N—C stretch of ribose-phosphate skeletal vibrations	Nucleic acids	
720–730	CH_2_ rocking	Lipids	

#### Lipids

The spectral region 3100–2800 cm^−1^ (Figs. 3 and 4(a)) mainly corresponds to lipids and proteins. The most distinguishable spectral features of lipids and phospholipids originate from the stretching vibration modes of the chain methylene (CH_2_) and olefinic (=CH) groups (3100–2800 cm^−1^). Fig. 4(a) shows the presence of 3010 cm^−1^ band assigned to olefinic groups of lipids and unsaturated fatty acids. The band at 3010 cm^−1^ was predominantly observed in both adipose tissues. A typical WAT adipocyte contains a large single lipid droplet stored in it. This lipid droplet consists of triglycerides. BAT adipocytes also contain triglycerides as multiple small vacuoles. Our data showed that WAT contains much higher unsaturated lipid content as compared to BAT. Interestingly, the =CH band was absent in both lung and liver tissues. The bands corresponding to CH_2_ groups are mainly the result of the saturated chains in lipids whereas the methyl (CH_3_) bands are attributed to the methyl groups in lipids, proteins, and nucleic acids. There are mainly four bands observed for lipids, attributed to the asymmetric and symmetric stretching vibrations of CH_2_ and CH_3_ groups that are found at ∼2960 (ν_as_CH_3_), ∼2923 (ν_as_CH_2_), 2870 (ν_s_CH_3_), and 2950 cm^−1^ (ν_s_CH_2_), respectively. CH_2_ and CH_3_ bands are greatly increased as compared to the rest of the peaks in 1800–875 cm^−1^ region for both BAT and WAT indicating the presence of higher lipid content. Furthermore, the relative position of ν_s_CH_2_ is an indicator of order or disorder of the lipid membrane which is dependent on the composition, content of membrane protein and other factors [13]. The band at 1745 cm^−1^ is attributed the carbonyl (νC=O) stretching band of triglycerides and cholesterol esters, see Fig. 4(b). Both BAT and WAT show considerably higher C=O band whereas liver and lung exhibit relatively low band at ∼1745 cm^−1^. The peak at 1456 cm^−1^ is mainly attributed to the CH_2_ bending mode of lipids which is prominent in all four tissues, see Fig. 4(b). All tissues show a peak in the range of 1158–1173 cm^−1^ which is assigned to the ν_as_CO—O—C vibration of cholesterol esters.

#### Proteins

Proteins are one of the major constituents in all tissues, thus their vibration bands are easily observed in FTIR microspectroscopy. Protein bands are predominantly observed in the 1800–1500 cm^−1^ spectral region (Figs. 2 and 3(b)). The infrared spectra of proteins exhibit two major bands, the amide I and amide II. These two amide bands are positioned at approximately 1665 cm^−1^ and 1555 cm^−1^. These are most widely used in studies on protein secondary structures and assigned primary to C=O stretching and NH bending modes. The amide I band is the strongest absorption band for proteins and is observed between 1623 and 1637 cm^−1^. The amide I band position and shape is the most sensitive to the secondary structure of proteins and structural configuration [14]. Amide I mode is extensively used to quantify and identity changes associated with α-helical (1648–1670 cm^−1^), β-plated sheet (1623–1637 cm^−1^), β-turns, and anti-parallel β-plated sheet (1670–1695 cm^−1^) structures of proteins [14], [15]. The major amide peak at ∼ 1656 cm^−1^ represents the α-helical structure of proteins. Both liver and lung tissues show a small shoulder at 1630 cm^−1^ corresponds to β-plated sheet structures. The amide I peak is clearly observed in lung and liver tissues in contrast to BAT and WAT tissues. Interestingly, the amide I band in WAT is much weaker in intensity as compared to the other three tissues, this is mainly due to the presence of high lipid content. This peak is slightly shifted in lung and liver tissues, see Fig. 3. The changes in the amide I band reflect the differences in strengths of the hydrogen bonding networks, coupling between dipoles, both inter and intra molecular, which subsequently provide amide I band its sensitivity to secondary conformational changes [14], [16]. The peak observed in the region of 1383–1395 cm^−1^ is attributed to the ν_s_COO- stretching mode of the side chains of amino acid residues of proteins, see Fig. 4(b). The major band shown in the range of 1312–1340 cm^−1^ is assigned to the amide III band of proteins, which was observable in all tissues. Proteins also exhibit a triplet band at approximately 1206, 1235 and 1280 cm^−1^, however, only 1235 and 1280 cm^−1^ bands are observed for liver and BAT tissue. Two bands observed at approximately 1206 and 1280 cm^−1^ do not show in lung and WAT tissues.

#### Other macromolecules

The bands observed in the spectral region of 1475–950 cm^−1^ (Fig. 3(b)) correspond to the bands from several macromolecules: carbohydrates, proteins, and nucleic acids (DNA and RNA). DNA, RNA, phosphorylated proteins, and phospholipids exhibit two bands at approximately 1231–1234 cm^−1^ and 1086–1097 cm^−1^ attributed to ν_as_PO_2_- and ν_s_PO_2_-, respectively [8]. These bands are observable in all tissues. Nucleic acids also show a specific band at 965 cm^−1^, which is shown in all tissues. The spectral region of 1150–1000 cm^−1^ corresponds to bands from carbohydrates. Three characteristic peaks at 1029, 1080, and 1150 cm^−1^ are observed for glycogen [17]. However, only the 1029 cm^−1^ band was observed in all tissues. The glycogen band at 1080 cm^−1^ may often overlap with the nucleic acid bands. The carbohydrate band observed at 1050 cm^−1^ was only prominent in WAT and liver tissues.

### Additional information

Tissue sections for FTIR imaging can be produced from fixed tissues embedded into paraffin or using cryo-sectioning. Chemical fixatives have been broadly used in preparing histological sections for microspectroscopic imaging [18]. The traditional chemical fixation of histological samples involves formalin fixation, decalcification, dehydration with ascending series of alcohol and xylene followed by infiltration with paraffin [19]. Paraffin embedding provides the necessary support for the histological sections, thus sections are easier to cut. Although, formalin-fixed paraffin-embedded (FFPE) samples provide a considerable amount of information regarding the biochemical nature and pathological state, chemical fixation is also associated with several drawbacks in FTIR microspectroscopic imaging. Chemical fixatives form chemical cross-links to proteins structures to preserve the tissue integrity. However, they could also alter the chemical composition of proteins and carbohydrates and also loss of lipids and some solubilized proteins from the sample. Specifically, xylene treatment is greatly responsible for reduction of phospholipid content of adipose tissue samples [20]. Another main source of error associated with FFPE samples is that the residual traces of paraffin which are evident even after chemical deparaffination [21]. Moreover, paraffin shows strong vibration bands in FTIR, making spectral interpretation more difficult. Furthermore, chemical dewaxing is time consuming and reagent consuming procedure. As a result, cryo-sectioning of fresh and frozen tissue is generally preferred over chemical fixation for molecular based investigations in FTIR microspectroscopic imaging [22]. Cryosectioning does not involve any fixation, labeling or any dehydration steps. Interestingly, this method circumvents the use of solvents that causes degradation or loss of some cellular components. Cryo-sectioning followed by FTIR microspectroscopic imaging provides spectroscopic information about lipid content that is largely lost through FFPE procedure [20]. Compared to chemical fixation, cryo-sectioning is a rapid process which requires minimal sample preparation. In general, tissue is prepared and embedded in a slurry of inert medium such as optimal cutting temperature (OCT) compound. Subsequently, the tissue sample is frozen in either isopentane sitting in dry ice or liquid nitrogen and sectioned with cryostat. One of the major issues with cryo-sectioning is that the lack of cross-linking agent makes the thin sections pliant, consequently sections wrinkle easily during the preparation. Therefore, repeated sectioning is required in order to obtain good quality sections and consistent results.
